# Editorial: Emerging Roles of the Gut Microbiota in the Pathogenesis of Metabolic Disorders

**DOI:** 10.3389/fendo.2021.736371

**Published:** 2021-08-13

**Authors:** Isabel Moreno-Indias, Antonio Salgado-Somoza, Hamid el Azzouzi, Mora Murri

**Affiliations:** ^1^Unidad de Gestión Clínica de Endocrinología y Nutrición del Hospital Virgen de la Victoria, Instituto de Investigación Biomédica de Málaga (IBIMA), Málaga, Spain; ^2^Centro de Investigación Biomédica en Red de Fisiopatología de la Obesidad y la Nutrición (CIBERobn), Instituto de Salud Carlos III, Madrid, Spain; ^3^Independent Researcher, Neufchâteau, Belgium; ^4^Department of Molecular Genetics, Erasmus University Medical Center, Rotterdam, Netherlands

**Keywords:** microbiota, metabolic disorders (MDs), diabetes, metabolism, obesity, phenylketonuria (PKU)

The human gut is inhabited by a highly complex and metabolically active microbial ecosystem composed by trillions of members. The gut microbiota heavily influences host physiology through microbiota-derived molecules (1). Variations in its composition induce metabolic changes that may result in alterations of host phenotype, being its metabolic activity essential in maintaining host homoeostasis and health. Moreover, gut microbes can also lead to severe metabolic disorders. On the other hand, gut microbiome is highly sensitive and can be altered throughout lifespan mostly by environmental factors. The host can affect the microbial ecosystem through its immune system, genetic background, sex, and age. All these factors may induce gut microbiota imbalances that are often associated with metabolic alterations.

Metabolic disorders are defined as a group of diseases where normal metabolic processes are disrupted due to the accumulation of large amounts of one metabolite or a deficiency of one or more metabolites. Metabolic disorders have a variety of clinical presentations ranging from acute symptoms in the neonatal period to slower, more gradual onsets at a later age. Metabolic diseases can be inherited or acquired during the lifetime. Inherited metabolic disorders result from a genetic defect in functioning of an intermediate metabolic pathway, while acquired disorders are resulting from external factors, with lifestyle factors as main causes.

The present Research Topic highlights the interplay between host metabolism and gut microbiota. Overall, it focuses on microbiota composition, functionality and metabolites in the pathogenesis of metabolic disorders and on the effect of microbiota modulation on host metabolism.

In recent years, a substantial body of literature has provided evidence for the link between gut microbiota and diabetes, disease that affects more than 463 million people worldwide (2). Several articles in the present topic focus on the relation between gut microbiota and the most prevalent type of diabetes, type 2 diabetes (T2D). A large cohort of T2D patient study presented by Diener et al. provides a potential set of four bacterial genera as a biomarker of T2D disease progression and risk. Moreover, they found that the four identified genera returned to near-normal levels in T2D treated individuals. In this line, a systematic review by Cao et al. analysed the reciprocal interactions between gut microbiota and anti-hyperglycemic drugs, focusing on the effect of the gut microbiome on diabetic control *via* bug-host interactions. They found that changes in specific taxa and β-diversity of gut microbiota were associated with metformin and acarbose in humans. A review by Huda et al. reports the recent findings regarding the role of gut microbiota in T2D, focusing on the causal relationship between microbiota and T2D. They not only summarized the associations between T2D and microbial metabolites but also described how host genetic architecture and the epigenome influence the microbial composition. Finally, they discussed future directions in this field, pointing at the potential of faecal microbiota transplantation, prebiotics, and probiotics supplementation as therapeutics for T2D. One of the most frequent and severe microvascular complications of diabetes is diabetic kidney disease. Fang et al. summarized in their review the current findings regarding the role of gut microbial metabolites in the development and progression of diabetic kidney disease.

Another type of diabetes, gestational diabetes mellitus (GDM) affects around 7–10% of all pregnancies worldwide (3). Soderborg et al. suggest a potential influence of GDM alone and together with maternal overweight/obesity on infant microbiota in patterns that set the stage for future risk of inflammatory and metabolic disease. Moreover, An et al. have explored the problem of small-sized new-borns finding that gut microbiota may play a role on metabolic disorders during this critical period, particularly through *Lactobacillus* and short-chain fatty acids (SCFAs).

The review of Martín-Núñez et al. compiled the latest evidence from human studies on the influence of *Helicobacter pylori* infection and its eradication therapies on the composition of the gut microbiota and host’s metabolic health. The effect of altered microbiota on metabolic disorders is clearly shown in several studies, however, it is intriguing that modulating metabolism can itself influence the composition of microbiota in the gut. Huang et al. showed in their article the effects of a 6-week program of training exercise and dietary restriction on gut microbiome composition, metabolism and central hemodynamic parameters in obese adolescents. They have found that exercise and diet interventions significantly reduced body weight, levels of glucose, triglycerides and HDL cholesterol, and improved measures of central hemodynamics, which correlated with altered gut microbiota. From a different point of view, Massier et al. reviewed the underlying mechanisms of an impaired intestinal barrier and its possible impact on metabolic health. They focus on recent findings on how endotoxemia and translocation of bacteria, bacterial genetic material and products may cause host dysfunction subsequently contributing to metabolic diseases. The authors concluded that it seems unavoidable that microbiota also contribute to the modulation of their metabolic environment shaping the body’s responses to nutrients and contributing ultimately to disease. This latter conclusion is of immense importance in the design and application of future therapies as inadequate research of interventional drug therapies could fire back through undesired modulation of the metabolic environment or the compounds produced by the microbiota itself.

The example of an inherited metabolic disease has been represented by the work by van der Goot et al. who used the metabolic disease phenylketonuria to discuss how microbial ecology and eco-evolutionary aspects within a challenged gastrointestinal tract lead to microbial relationships that can be used to prevent neurological problems through the fascinating gut-brain axis.

Within the scientific community there is no doubt about the fact that alterations in gut microbiota constitute an important factor in the development of metabolic disorders. Moreover, current lifestyles in the developed countries are foreboding that metabolic disorders due to deranged gut microbiota will not decrease any time soon. Therefore, understanding the role of gut microbiota in the pathogenesis of metabolic disorders may hold exciting prospects for the treatment of these diseases. However, the interplay is still poorly understood which makes real breakthroughs in therapeutic interventions less feasible. Taken together, to delineate the exact mechanisms of this interplay, different research fields must combine their expertise to tackle this important question.

## Author Contributions

IM-I, AS-S, HA, and MM wrote and edited the editorial. All authors contributed to the article and approved the submitted version.

## Funding

IM-I is supported by Miguel Servet I program (CP16/00163) from ISCIII and co-funded by FEDER funds. IM-I is also supported by PI15/01114, PI18/01160 Madrid, Spain, and by CB06/03/0018. MM is supported by Miguel Servet I program (CP17/00133) from ISCIII and co-funded by FEDER funds. MM is also supported by UMA18-FEDERJA-285 co-funded by Malaga University, Junta de Andalucıa, and FEDER funds, CB06/03/0018 and PI-0297-2018 co-funded by FEDER funds and Consejerıa de Salud y Familia, Junta de Andalucıa, and by the grant PI19/00507 from ISCIII co-funded by FEDER funds, Spain.

## Conflict of Interest

The authors declare that the research was conducted in the absence of any commercial or financial relationships that could be construed as a potential conflict of interest.

## Publisher’s Note

All claims expressed in this article are solely those of the authors and do not necessarily represent those of their affiliated organizations, or those of the publisher, the editors and the reviewers. Any product that may be evaluated in this article, or claim that may be made by its manufacturer, is not guaranteed or endorsed by the publisher.

## References

[1] Moreno-Indias I Cardona F Tinahones FJ Isabel Queipo-Ortuño M. Impact of the Gut Microbiota on the Development of Obesity and Type 2 Diabetes Mellitus. Front Microbiol (2014) 5:190. 10.3389/fmicb.2014.00190 PMC4010744 24808896

[2] Saeedi P Petersohn I Salpea P Malanda B Karuranga S Unwin N. Global and Regional Diabetes Prevalence Estimates for 2019 and Projections for 2030 and 2045: Results From The International Diabetes Federation Diabetes Atlas, 9th Edition. Diabetes Res Clin Pract (2019) 157:107843. 10.1016/j.diabres.2019.107843 31518657

[3] Behboudi-Gandevani S Amiri M Bidhendi Yarandi R Ramezani Tehrani F. The Impact of Diagnostic Criteria for Gestational Diabetes on Its Prevalence: A Systematic Review and Meta-Analysis. Diabetol Metab Syndr (2019) 11:11. 10.1186/s13098-019-0406-1 30733833 PMC6359830

